# The Lowe Syndrome Protein OCRL1 Is Required for Endocytosis in the Zebrafish Pronephric Tubule

**DOI:** 10.1371/journal.pgen.1005058

**Published:** 2015-04-02

**Authors:** Francesca Oltrabella, Grzegorz Pietka, Irene Barinaga-Rementeria Ramirez, Aleksandr Mironov, Toby Starborg, Iain A. Drummond, Katherine A. Hinchliffe, Martin Lowe

**Affiliations:** 1 Faculty of Life Sciences, University of Manchester, Manchester, United Kingdom; 2 Nephrology Division, Massachusetts General Hospital and Department of Genetics, Harvard Medical School, Charlestown, Massachusetts, United States of America; University of Pennsylvania School of Medicine, UNITED STATES

## Abstract

Lowe syndrome and Dent-2 disease are caused by mutation of the inositol 5-phosphatase OCRL1. Despite our increased understanding of the cellular functions of OCRL1, the underlying basis for the renal tubulopathy seen in both human disorders, of which a hallmark is low molecular weight proteinuria, is currently unknown. Here, we show that deficiency in OCRL1 causes a defect in endocytosis in the zebrafish pronephric tubule, a model for the mammalian renal tubule. This coincides with a reduction in levels of the scavenger receptor megalin and its accumulation in endocytic compartments, consistent with reduced recycling within the endocytic pathway. We also observe reduced numbers of early endocytic compartments and enlarged vacuolar endosomes in the sub-apical region of pronephric cells. Cell polarity within the pronephric tubule is unaffected in mutant embryos. The OCRL1-deficient embryos exhibit a mild ciliogenesis defect, but this cannot account for the observed impairment of endocytosis. Catalytic activity of OCRL1 is required for renal tubular endocytosis and the endocytic defect can be rescued by suppression of PIP5K. These results indicate for the first time that OCRL1 is required for endocytic trafficking in vivo, and strongly support the hypothesis that endocytic defects are responsible for the renal tubulopathy in Lowe syndrome and Dent-2 disease. Moreover, our results reveal PIP5K as a potential therapeutic target for Lowe syndrome and Dent-2 disease.

## Author Summary

Phosphoinositide lipids are key regulators of cellular physiology and consequently enzymes that generate or remove these lipids are of fundamental importance. Mutation of one such enzyme, called OCRL1, causes two disorders in humans, Lowe syndrome and Dent-2 disease. However, the underlying mechanisms remain poorly defined. Here, we demonstrate that OCRL1 regulates endocytosis, the process by which cells internalize material from their extracellular environment. Importantly, this is demonstrated in a physiologically relevant tissue in vivo, namely the zebrafish renal tubule. Defective endocytosis can explain the renal symptoms seen in Lowe syndrome and Dent-2 patients. We also report that defects in cell polarity or cilia formation cannot explain the renal symptoms. This study not only increases our understanding of the endocytic pathway, it also provides a mechanistic explanation for the renal defects observed in Lowe syndrome and Dent-2 patients.

## Introduction

Oculorecerbrorenal syndrome of Lowe is a rare X-linked disorder with the hallmark symptoms of congenital cataracts, mental retardation and proximal renal tubulopathy [1]. Lowe syndrome is caused by mutation of the gene encoding OCRL1, an inositol 5-phosphatase which preferentially hydrolyses PtdIns(4,5)P_2_, although it also displays activity towards PtdIns(3,4,5)P_3_ [2]. OCRL1 has a modular domain structure, with an N-terminal pleckstrin homology domain, a central 5-phosphatase, and C-terminal ASH and Rho-GAP like domains [3]. OCRL1 is localised to several cellular compartments including the *trans*-Golgi network [4], early endosomes [5,6,7,8], clathrin-coated pits and vesicles [5,6,7,9], as well as cellular junctions in polarised cells [10], lamellipodia in migrating cells [11,12], phagosomes in phagocytic cells [13,14], and the intercellular bridge in cells undergoing cytokinesis [15]. More recently, OCRL1 has also been localised to the cilium [16,17]. Membrane targeting of OCRL1 is mediated by Rab GTPases, which bind to the ASH domain [18], while additional targeting information is conferred by binding to the vesicle coat proteins clathrin and α-adaptin [5,6,9,19], Rac1 [11], and the endocytic proteins APPL1 and IPIP27A and B, also known as Ses1 and 2 [7,20,21]. In line with its varied cellular locations, OCRL1 has been implicated in a number of cellular processes. These include trafficking within the endocytic pathway [6,7,8,9,22,23], phagocytosis [13,14], cell migration and polarity [10,12], intracellular signalling, cytokinesis [15,24] and ciliogenesis [16,17,25]. OCRL1 may regulate these diverse processes through altering actin dynamics at various cellular compartments [26].

Mutation of OCRL1 also causes Dent-2 disease, which has a similar renal phenotype to Lowe syndrome, but milder eye and neurological defects [27]. The renal tubular dysfunction in both disorders is invariably manifest as low molecular weight proteinuria, with more variable degrees of hypercalciuria, aminoaciduria, and renal tubular acidosis [27]. How loss of OCRL1 leads to the renal pathology seen in Lowe syndrome and Dent-2 disease is currently unclear. It has been proposed that defective endocytic trafficking may be responsible, with impaired trafficking of the multi-ligand receptor megalin (also called LRP2), which is responsible for the majority of protein reabsorption in the proximal tubule, leading to the proteinurea observed in these conditions [28]. However, it has also been proposed that defects in cellular junction formation resulting in loss of cell polarity within the renal tubular epithelium may be the underlying cause of the renal tubulopathy [10]. Alternatively, several recent papers have proposed that defective ciliogenesis is the cause of the symptoms of Lowe syndrome and Dent-2, and suggested that these disorders may be classified as ciliopathies [16,17,25].

Until recently there was no animal model to investigate the disease mechanisms of Lowe syndrome and Dent-2. In mice, the related inositol 5-phosphatase Inpp5b can fully compensate for loss of OCRL1, with no detectable phenotype in the knockout animal [29]. This issue was overcome by humanizing the Inpp5b locus, which resulted in mice that display both proteinuria and aminoaciduria, although the underlying mechanisms were not explored in this study [30]. As an alternative strategy we recently generated a zebrafish model for Lowe syndrome with a retroviral insertion in the OCRL1 locus to stably attenuate gene expression [31]. This model recapitulates several of the neurological features of Lowe syndrome including increased propensity to undergo seizures and the presence of cystic brain lesions. We now demonstrate that endocytosis within the pronephros, the embryonic renal tubule, is impaired in OCRL1-deficient zebrafish. We also observe a reduction in levels of megalin protein and its mislocalization in the endocytic pathway, together with altered morphology and abundance of endocytic compartments, indicative of defective endocytic trafficking. These effects are not due to ciliary defects, and cell polarity is normal in the zebrafish model. Our results reveal for the first time that OCRL1 is required for efficient endocytosis in vivo, and strongly support the hypothesis that endocytic defects give rise to the renal manifestations of Lowe syndrome and Dent-2 disease.

## Results

### Endocytosis in the pronephric tubule is impaired in OCRL1 deficient zebrafish embryos

**Fig 1:**
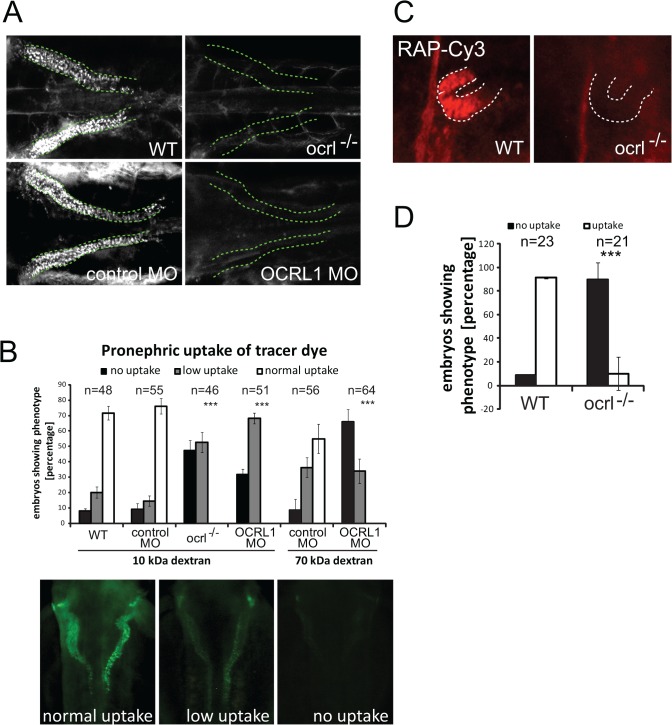
Impairment of pronephric uptake in OCRL1 deficient zebrafish embryos. A. Confocal images of wild-type (WT), *ocrl^-/-^* mutant, control morphant or OCRL1 morphant 72 hpf zebrafish embryos that were injected with Alexa 488-10 kDa dextran (white) and imaged after 2.5 h. The pronephric tubules are indicated with a green dashed line. B. Top: Quantification of pronephric uptake of 10 kDa (2.5 h) or 70 kDa dextran (4 h) in control, *ocrl^-/-^* mutant and morphant embryos. Bottom: Representation of normal, low and no dextran uptake in injected. C. Wild-type (WT) and *ocrl^-/-^* mutant embryos were injected with RAP-Cy3 (red) and pronephric accumulation after 60 min monitored by fluorescence microscopy. D. Quantification of pronephric uptake of RAP-Cy3 in control and *ocrl^-/-^* mutant embryos. Data are presented as the mean ± SD. Statistical analysis was performed using the Pearson’s chi-squared test. ***p < 0.0001.

To investigate endocytosis in the OCRL deficient zebrafish we used an established assay in which fluorescent endocytic tracer is injected into the cardinal vein, followed by filtration and reabsorption into the cells lining the pronephric tubule [32]. Endocytic uptake into the renal tubular cells is then monitored by fluorescence microscopy. We initially used fluorescent 10kDa dextran as tracer, which was present throughout the vasculature immediately following injection, and efficiently taken up by the renal tubular cells of control embryos (S1A Fig.). The dextran was present in puncta that correspond to endocytic compartments (Figs. 1A and S1B). In contrast, uptake was severely compromised in the transgenic OCRL1-deficient embryos, herein referred to as *ocrl^-/-^*, or embryos that had previously been injected with a translation blocking OCRL1 morpholino to deplete OCRL1 [31] (Fig. 1A and B, and S2). Similar results were seen with 70 kDa fluorescent dextran, which is also filtered and reabsorbed in control embryos, but poorly taken up by the pronephros of OCRL1 morphants (Fig. 1B). We also investigated uptake of receptor–associated protein (RAP), which can be used as a ligand for zebrafish megalin [33]. As shown in Fig. 1C and D, RAP uptake was also impaired in the *ocrl^-/-^* embryos, indicating defective megalin-dependent endocytosis upon loss of OCRL1.

A possible explanation for the reduced endocytic uptake in the pronephros of OCRL1-deficient embryos is that development of the organ itself is affected. We therefore analysed morphology of the pronephros in transgenic embryos expressing a GFP proximal tubule reporter (33D10-GFP) [34]. Morpholino knockdown of OCRL1 had no obvious effect on the organisation of the proximal pronephric tubule (S3 Fig.). Similar results were obtained in embryos expressing GFP in the pronephric tubule under the control of the enpep promoter [35] (S3 Fig.). We also labelled embryos with the 3G8 antibody that marks the pronephric brush border. Again, pronephros morphology was found to be the same in *ocrl^-/-^* embryos and controls (S3 Fig.).

Both Lowe syndrome and Dent-2 disease display a clear renal tubulopathy [27]. However, there have been reports of glomerular dysfunction in patients, resulting in loss of the filtration barrier and nephrotic syndrome [36,37]. Whether this is a direct effect or a downstream consequence of tubular dysfunction is currently unclear. We therefore analysed glomerular filtration in the *ocrl^-/-^* mutant by injecting 500 kDa dextran, which is too large to pass through a normally functioning glomerulus, and monitoring its loss from the embryos over time. As shown in S4 Fig., the 500 kDa dextran was retained to a similar degree in both control wild-type and *ocrl^-/-^* mutant embryos, indicating an intact filtration barrier in the mutant. Thus, while the mutant embryos display a tubular uptake defect, the functioning of the glomerulus is unaffected.

### Megalin abundance and subcellular distribution are altered in OCRL1 deficient embryos

**Fig 2:**
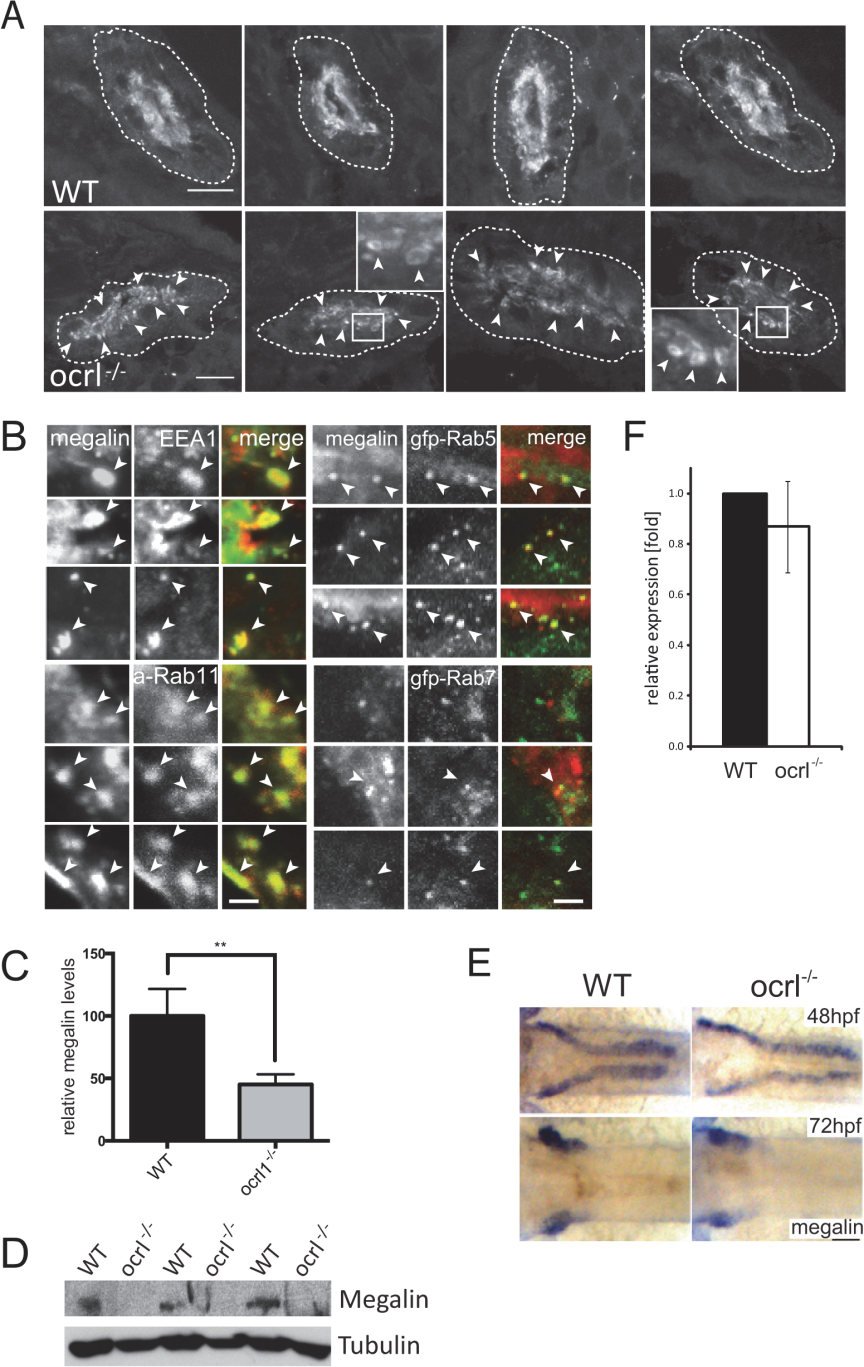
Megalin transcript and protein analysis in OCRL1-deficient zebrafish embryos. A. Transverse confocal images of the proximal pronephric region of wild-type (WT) and *ocrl^-/-^* mutant 72 hpf embryos labelled with anti-megalin antibodies. The white dashed lines indicate the outline of pronephric tubules. Arrowheads indicate sub-apical punctate and vacuolar megalin staining. B. Transverse confocal images of the proximal pronephric region of 72 hpf *ocrl^-/-^* embryos labelled with antibodies to megalin (green in left panel, red in right panel) and EEA1 (red) or GFP (gfp-, green) to detect ectopically expressed Rab5 or Rab7. mApple (a-) tagged Rab11 is in red. Arrowheads indicate colocalisation. C. Quantification of the relative fluorescence levels of megalin in confocal transverse sections of the indicated embryo types. D. Western blot of 72 hpf wild-type (WT) or *ocrl^-/-^* embryos with antibodies to megalin and tubulin. Three equivalent samples for genotype are analyzed. E. In situ hybridisation of megalin transcript in 48 hpf (top) and 72 hpf (bottom) wild-type (WT) or *ocrl^-/-^* embryos. F. Quantitative RT-PCR (qPCR) of megalin transcript levels in wild type and *ocrl^-/-^* embryos at 72 hpf. Data are presented as the mean ± SD. Statistical analysis was performed using the unpaired t-test. ***p < 0.0001. Scale bars in A, B and E represent 10, 2 and 20 μm respectively.

It has been hypothesised that defective endocytic trafficking of the multi-ligand receptor megalin could explain the proteinurea seen in Lowe syndrome and Dent-2 disease [28]. Similarly, the endocytic defect we observe could arise from defective megalin trafficking, either due to a reduced rate of internalization from the plasma membrane, or reduced recycling from apical endosomes in turn leading to a deficit at the plasma membrane and consequently reduced endocytosis. To investigate these possibilities we performed megalin staining in wild-type and *ocrl^-/-^* embryos. Megalin is expressed in the proximal segment of the pronephric tubule of both embryo types, and the same was true for OCRL1 morpholino injected embryos (S5A Fig.). Analysis of cryosections revealed that megalin was correctly targeted to the apical pole in control and OCRL1-deficient embryos, arguing against a defect in cell polarity in the renal tubular cells upon loss of OCRL1 (Figs. 2A and S5B). Consistent with this, NaK ATPase is restricted to the basolateral membrane of pronephric cells of both control and *ocrl^-/-^* embryos (S5B Fig.). Moreover, by electron microscopy, the organisation of the pronephric tubular epithelium is normal in *ocrl^-/-^* embryos, with a clear apical brush border and intercellular junctions that are correctly positioned and morphologically indistinguishable from those in control embryos (S6 Fig.). These results argue against a defect in cell polarity or intercellular junction formation as the cause of renal tubular defects in Lowe syndrome and Dent-2 disease.

Although megalin was correctly targeted to the apical pole in the OCRL1-deficient embryos, closer inspection revealed localization to cytoplasmic puncta and vacuolar compartments in close proximity to the brush border (Fig. 2A). This contrasted with control embryos, where megalin was predominantly localized to the apical brush border. The megalin positive compartments in *ocrl^-/-^* embryos resembled sub-apical endosomes, which was further analyzed by double-labelling with various endocytic markers. As shown in Fig. 2B, the punctate and vacuolar megalin colocalized with endogenous EEA1 and ectopically expressed GFP-Rab5, markers of early endosomes, and GFP-Rab11, which marks recycling endosomes, with a lower degree of overlap with the late endosome marker GFP-Rab7. Thus, megalin is partially redistributed from the brush border to sub-apical early and recycling endosomes in *ocrl^-/-^* embryos. This is most consistent with a defect in endocytic recycling, resulting in less megalin at the plasma membrane and consequently reduced endocytosis.

As well as altered megalin distribution in *ocrl^-/-^* embryos, we also observed a reduction in megalin abundance. Quantitation of fluorescence intensity revealed a 50% reduction in megalin protein levels in the mutant embryos (Fig. 2C). Reduction in megalin abundance was also observed by Western blotting (Fig. 2D). This was not due to changes in expression of the megalin mRNA. Megalin transcript was correctly localised within the pronephros at both 48 and 72 hpf in mutant embryos (Fig. 2E), and quantitative PCR at 72hpf indicated that the levels of megalin mRNA were the same as in control embryos (Fig. 2F). The reduced abundance of megalin protein in the absence of altered transcript levels is consistent with mis-trafficking of the protein within the endocytic pathway, similar to that observed in CLC5 knockout mice that fail to efficiently recycle megalin from endosomes to the apical plasma membrane [38,39].

### OCRL1 deficiency results in reduced amounts of endocytic apparatus in the pronephric tubule

**Fig 3:**
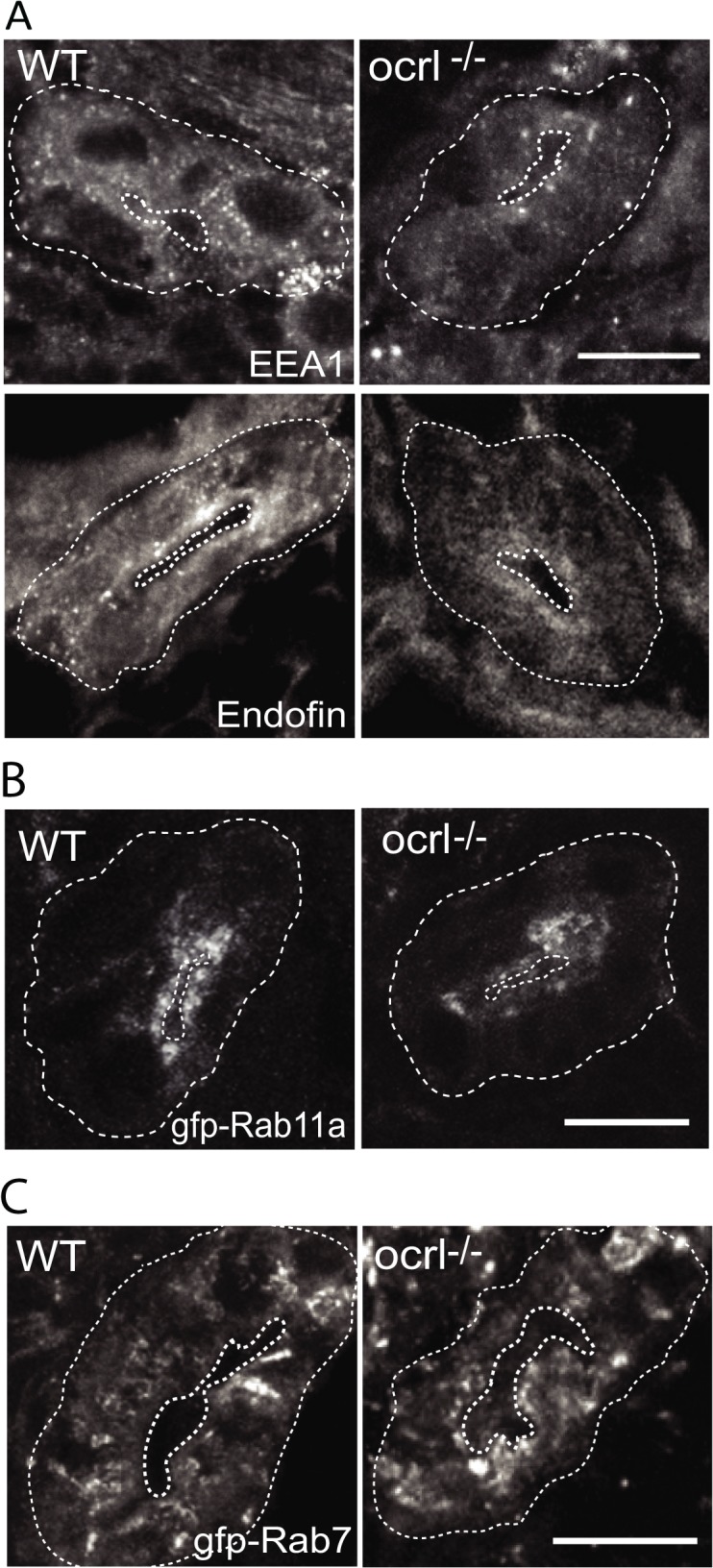
Reduced endosomal staining in OCRL1 deficient pronephros. A-C. Confocal transverse sections of the zebrafish proximal pronephric tubule of 72 hpf wild-type (WT) and *ocrl^-/-^* mutant embryos labelled with antibodies to EEA1 or endofin (A), or to GFP (B and C) to detect expressed GFP-Rab11 (B) or GFP-Rab7 (C). White dashed lines indicate the outline of pronephric tubules. Scale bars represent 10 μm.

It has previously been shown in both mouse and zebrafish that loss of megalin results in a profound reduction in abundance of endocytic compartments in the renal proximal tubule, likely due to reduced flux of material through the endocytic pathway [33,40,41]. We therefore performed fluorescence microscopy to visualise endocytic compartments in the proximal tubular cells of *ocrl^-/-^* embryos, which have reduced endocytosis and lower megalin abundance in comparison to controls (Figs. 1 and 2). Early endosomes, labelled with antibodies to EEA1 or endofin, were abundant in the apical pole in control embryos (Fig. 3A). Although the distribution of EEA1 and endofin in *ocrl^-/-^* embryos was similar to controls, the labelling intensity was weaker (Fig. 3A). This was also true for Rab11, which marks recycling endosomes (Fig. 3B), while late endosomes, labelled with GFP-Rab7, were not obviously affected (Fig. 3C).

**Fig 4:**
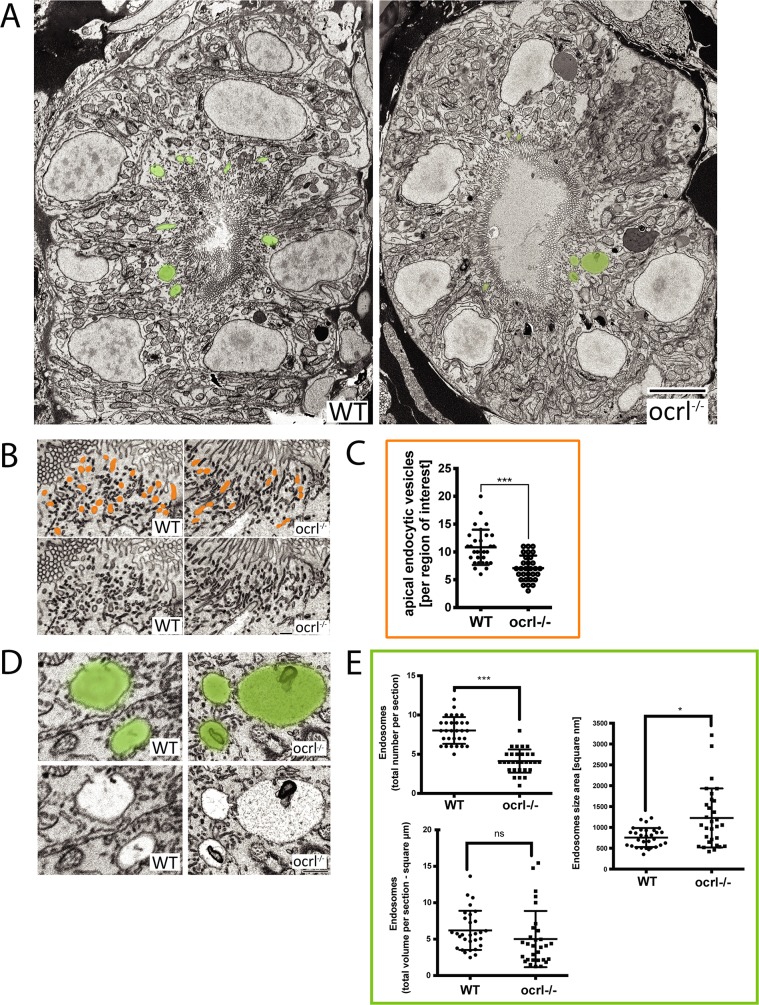
Electron microscopy analysis of endocytic compartments in OCRL1 deficient pronephros. A. Block face scanning electron microscopy (SEM) images of transverse sections through the zebrafish proximal pronephric tubule of wild-type and *ocrl^-/-^* mutant 72 hpf embryos. The apical membrane, identified by numerous microvilli, lines the central lumen of the pronephric tubule. Vacuolar endosomes are false coloured in green. B and D. Block face SEM showing apical endocytic vesicles at the apical pole of pronephric proximal tubule cells (false coloured in orange in top row) (B) and vacuolar endosomes (false coloured in green in top row) (D). C and E. Quantification of endocytic compartments. Numbers of apical endocytic vesicles were counted per region of interest (C), and vacuolar endosome number, size and total area were counted per entire section (E). Data are presented as the mean ± SD. Statistical analysis was performed using the unpaired t-test. ***p < 0.0001. Scale bars represent 5 μm (A), 2 μm (D) or 1 μm (B).

To analyse endocytic compartments at higher resolution and perform a more quantitative analysis we used electron microscopy. Endocytic compartments in the renal proximal tubule are well defined morphologically, comprising numerous sub-apical endocytic vesicles of less than 250 nm diameter, electron lucent larger vacuolar endosomes that are typically >0.5 μm diameter, and electron dense recycling tubules that mediate receptor recycling to the plasma membrane [42,43]. All of these compartments were observed in both control and *ocrl^-/-^* embryos (Fig. 4A, B and D). However, quantitation revealed that the numbers of both endocytic vesicles and vacuolar endosomes were significantly reduced in the *ocrl^-/-^* embryos (Fig. 4C and E). Moreover, while the morphology of endocytic vesicles appeared normal in *ocrl^-/-^* embryos (Fig. 4B), the vacuolar endosomes were larger compared to control embryos (Fig. 4A, D and E). A similar enlarged endosome phenotype was previously reported in OCRL1-depleted cultured cells [8], and is consistent with reduced membrane recycling from this compartment. Taken together, our immunofluorescence and electron microscopy experiments indicate that deficiency of OCRL1 within the renal tubule leads to a reduction in the number of early endocytic compartments, which is accompanied by changes in the size of vacuolar endosomes.

### The endocytosis defect in OCRL1 deficient embryos is independent of ciliogenesis

**Fig 5:**
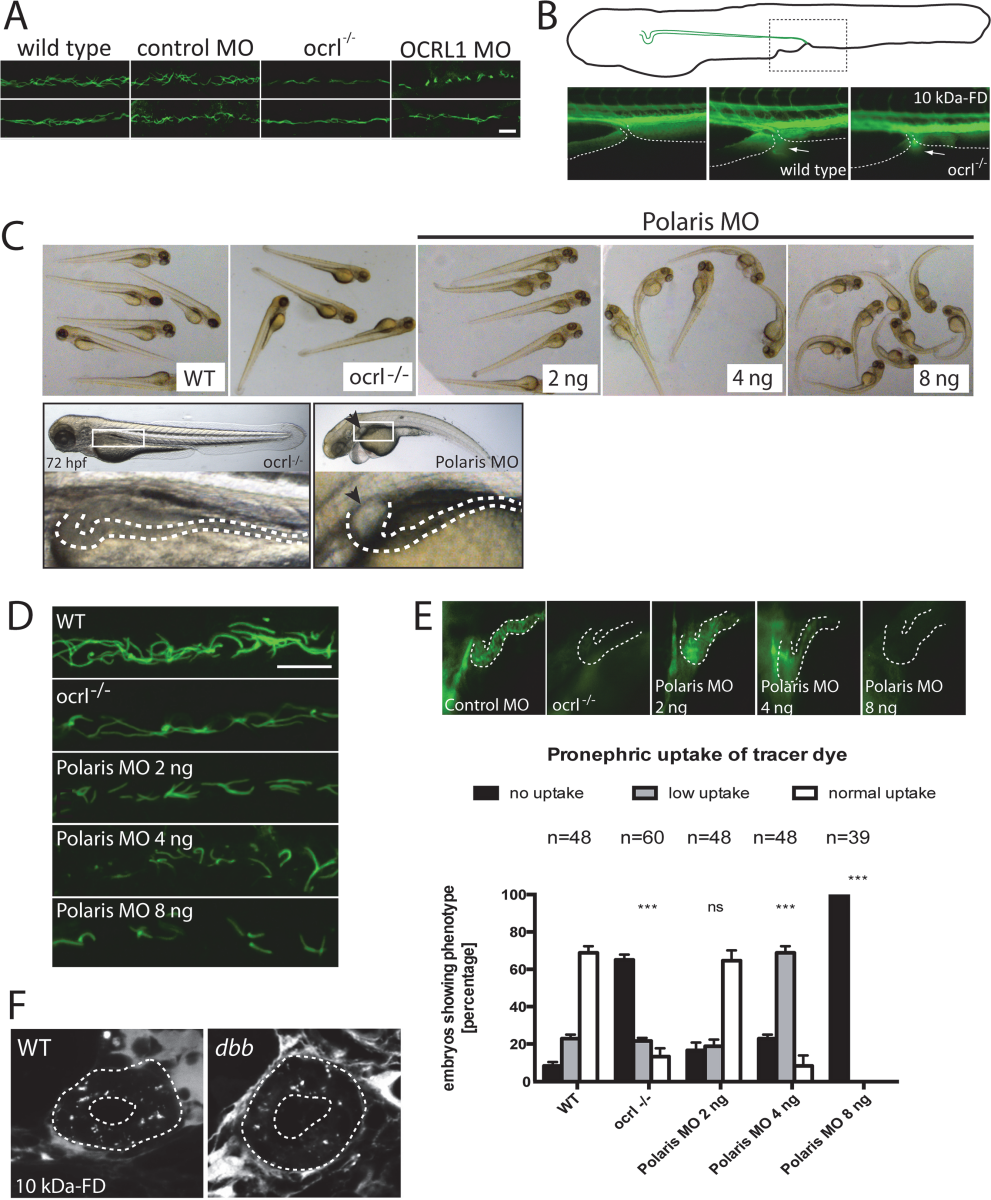
Pronephric cilia in *ocrl^-/-^* zebrafish. A. Confocal images of pronephric cilia, detected using anti-acetylated tubulin antibody, in wild-type, *ocrl^-/-^* mutant, control morphant or OCRL1 morphant zebrafish embryos (26hpf). B. Fluorescence dissecting microscope image of excretion of Alexa 488-10 kDa dextran from the cloacae of zebrafish embryos (72hpf). Bottom panels show cloacae immediately after injection (left) and excreting dextran 30–60s after injection (wild-type middle, *ocrl^-/-^* right). Dextran excretion was identical in control and *ocrl^-/-^* embryos (20 embryos of each genotype, 2 independent experiments). C. Brightfield images of wild-type (WT), *ocrl^-/-^* mutant or IFT88/polaris morphant (MO) embryos. The morphants were injected with different concentrations of morpholino as indicated. Embryos were imaged using brightfield microscopy. Bottom panel shows *ocrl^-/-^* mutant and polaris morphant (injected with 4 ng MO) and zoom of boxed area. The arrowhead indicates a pronephric cyst in the polaris morphant. D. Confocal images of pronephric cilia, detected using anti-acetylated tubulin antibody, in wild-type (WT), *ocrl^-/-^* mutant or IFT88/polaris morphant (MO) embryos. E. Wild-type (WT), *ocrl^-/-^* mutant and IFT88/polaris morphant embryos were injected with Alexa 488-10 kDa dextran (green) and pronephric accumulation after 2.5 h monitored by fluorescence microscopy. The pronephric tubules are indicated with a dashed line. Uptake was quantitated as indicated. Data are presented as the mean ± SEM. Statistical analysis was performed using the Pearson’s chi-squared test. ***p < 0.0001, **p < 0.001, *p < 0.01. F. Confocal transverse sections of the zebrafish proximal pronephric tubule of 72 hpf wild type and *double bubble (dbb*) cilia mutant showing 10 kDa-FD uptake into endocytic compartments in pronephric cells 2h after injection. Scale bars represent 10 μm (A and D).

Several studies have described defects in ciliogenesis upon OCRL1 depletion, and proposed that the symptoms of Lowe syndrome and Dent-2 disease are due to ciliary defects [16,17,25]. It has also recently been reported that loss of primary cilia in the renal tubule can reduce the rate of apical endocytosis due to impaired detection of fluid shear stress [44]. We therefore investigated whether effects upon ciliary function could account for endocytic defects we observe in the OCRL1 deficient embryos, possibly as a consequence of impaired fluid flow within the pronephric tubule. As previously reported, OCRL1-deficient embryos had shorter and fewer cilia, labelled using an antibody to acetylated tubulin, within the pronephros [16] (Fig. 5A), consistent with a role for OCRL1 in ciliogenesis. However, several lines of evidence indicate that impaired ciliary function cannot account for the endocytic defect observed in the OCRL1 deficient embryos. Firstly, we do not observe significant impairment of fluid flow within the pronephros of *ocrl^-/-^* embryos, as indicated by normal excretion of unabsorbed fluorescent dextran from the cloacae (Fig. 5B). In line with this, the mutant does not develop renal cysts (Fig. 5C). Secondly, we compared endocytosis in the *ocrl^-/-^* mutant with embryos injected with a morpholino against IFT88/*polaris*, which is required for ciliogenesis in the pronephric tubule [45]. Embryos injected with high doses of morpholino displayed characteristic features of defective ciliogenesis, with a curved body axis, oedema and dilation of the renal tubule (Fig. 5C). Notably, these phenotypes are absent from the *ocrl^-/-^* embryos (Fig. 5C). Consistent with the study of [44], renal endocytosis was impaired in the IFT88/*polaris* morphants. In embryos injected with lower doses of morpholino, a ciliogenesis defect was still evident, which was comparable to (at 2 ng morpholino) or more severe than (at 4 ng morpholino) that observed in the *ocrl^-/-^* mutant (Fig. 5D). Importantly, these IFT88/*polaris* morphants were still able to carry out endocytosis, indicating that the impaired endocytosis in the *ocrl^-/-^* mutant cannot be explained by a defect in ciliogenesis (Fig. 5E). Further support comes from the observation that the zebrafish cilia mutant *double bubble*, which also has a more severe ciliogenesis defect than the *ocrl^-/-^* line, can endocytose dextran [32,46] (Fig. 5F). Our results indicate that defects in dextran uptake and ciliogenesis are separable, and that loss of endocytic tracer uptake in the OCRL1 mutant and morphant embryos is not a downstream consequence of impaired ciliary function.

### OCRL1 5-phosphatase activity and interaction with trafficking machinery is required for endocytosis in the pronephric tubule

**Fig 6:**
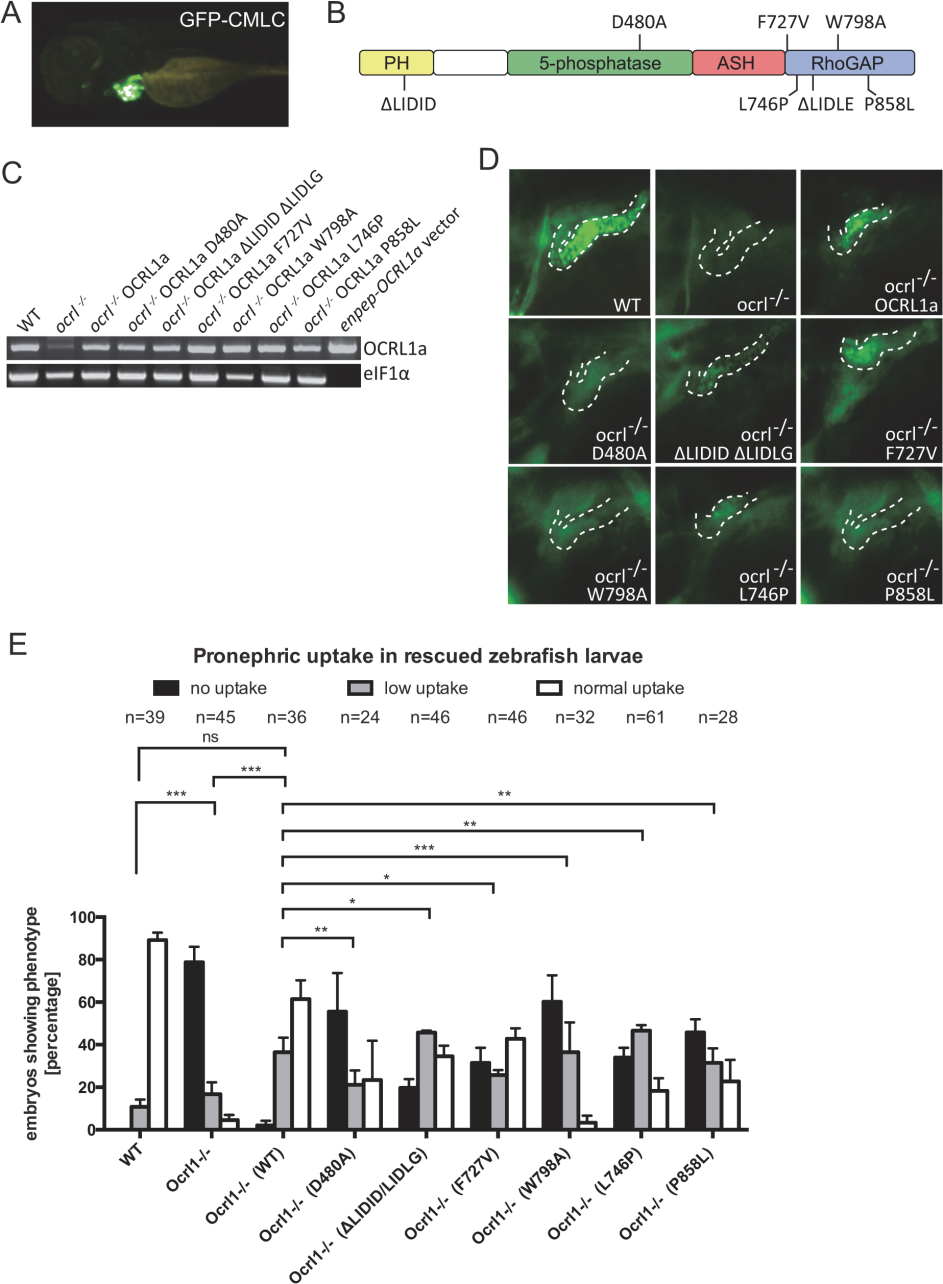
Rescue of the pronephric uptake defect in OCRL1 deficient embryos. A. Lateral view of a wild-type zebrafish embryo co-injected with *cmlc2*:GFP and *enpep*:OCRL1a. B. Schematic diagram of OCRL1 showing domain organization and localization of deletion and point mutants used in rescue experiments. C. RT-PCR detection of OCRL1 mRNA in WT, *ocrl^-/-^* embryos or *ocrl^-/-^* embryos expressing the indicated OCRL1a constructs. eIF1α was used as a control. The vector only lane is a positive control for the OCRL1 PCR, and lacks eIF1α. D. Images of pronephric uptake of Alexa 488-10 kDa dextran (green) in wild type (WT), *ocrl^-/-^* embryos or *ocrl^-/-^* embryos expressing OCRL1a, catalytically inactive GFP-OCRL1a (D480A), OCRL1a unable to bind clathrin (ΔLIDID ΔLIDLG), Rab GTPases (F727V), F&H domain proteins such as APPL1 and IPIP27A/B (W798A), or OCRL1a harbouring the Lowe syndrome mutation L746P or Dent-2 mutation P858L. The pronephric tubules are indicated with a dashed line. E. Quantification of pronephric uptake of Alexa 488-10 kDa dextran in each of the indicated embryo types. Data are presented as the mean ± SEM. Statistical analysis was performed using the Pearson’s chi-squared test. ***p < 0.0001, **p < 0.001, *p < 0.01.

To confirm the specificity of the renal endocytosis defect, and to dissect the mechanisms involved, we performed rescue experiments in which OCRL1 was transiently expressed in mutant embryos under the control of the enpep kidney-specific promoter [35]. We used untagged zebrafish OCRL1 for these experiments. In order to identify transduced embryos, we co-expressed GFP under the control of the cardiac myosin light chain 2 (cmlc2) promoter, which strongly labels the heart (Fig. 6A). Control experiments indicated that co-expression was efficient, with 75% of embryos expressing the cmlc2-GFP reporter also positive for enpep-GFP in the pronephros. In the case of OCRL1 (domain organization shown in Fig. 6B), we could confirm expression by RT-PCR (Fig. 6C). As shown in Fig. 6D, re-expression of OCRL1 in the pronephros of *ocrl^-/-^* embryos was able to restore dextran endocytosis. Quantitation revealed that of the cmlc2-GFP positive embryos co-expressing wild-type OCRL1, 60% had efficient (normal) dextran uptake, with an additional 35% displaying a lower degree of uptake (Fig. 6E). This compares favourably to wild-type embryos which have 80–90% high uptake, indicating an efficient rescue.

Having established that re-expression of OCRL1 can restore the endocytic function of pronephric cells, we next performed rescue experiments with mutant constructs deficient in either catalytic activity or interaction with various binding partners (Fig. 6B). All constructs were expressed using the enpep promoter and were expressed at equivalent levels (Fig. 6C). The mutations are exposed on the protein surface and are unlikely to induce protein misfolding and degradation, as determined from the crystal and NMR structures of OCRL1 and previous mutagenesis studies [3,9,19,20,21]. We are therefore confident that any changes in rescue ability are not due to differences in protein abundance. The catalytically inactive D480A mutant was unable to restore dextran uptake (Fig. 6D and E), indicating that 5-phosphatase is important for OCRL1 function in endocytic trafficking in the pronephric tubule. Expression of a mutant lacking both clathrin binding sites (ΔLIDID ΔLIDLG), or lacking binding to Rab GTPases (F727V, equivalent to F668V in human OCRL1) were also deficient in rescue, albeit to a lesser extent than the catalytically inactive D480A mutant (Fig. 6D and E). We next investigated whether interaction of OCRL1 with the F&H domain proteins plays a role in this process. The F&H domain proteins IPIP27A and B (Ses1 and 2) have previously been shown to regulate endocytic recycling in mammalian cells [21]. OCRL1 binds to IPIP27A and B (Ses1 and 2) and the other known F&H domain protein APPL1 through a conserved binding site present within a helical region of the Rho GAP-like domain, and mutation of this site abolishes binding to both APPL1 and IPIP27 [47]. Expression of zebrafish OCRL1 W798A, which is mutated in the F&H binding site, failed to rescue the endocytic defect in *ocrl^-/-^* embryos (Fig. 6D and E). The poor rescue efficiency of this construct was comparable to that of the catalytically inactive D480A mutant. This indicates the importance of OCRL1 binding to F&H domain proteins for its function in the endocytic pathway, and is consistent with the idea that impaired recycling underlies the endocytosis defect in the *ocrl^-/-^* mutant.

### Lowe syndrome and Dent-2 disease mutants fail to rescue the renal endocytosis defect of OCRL1-/- embryos

Zebrafish OCRL1 shares a high degree of sequence conservation with its human orthologue [31]. We exploited this fact to generate mutations in zebrafish OCRL1 that correspond to disease-causing mutations in humans. Interestingly, the human equivalent of the F727V mutation described above, which abolishes Rab binding [48], has been identified as a Lowe syndrome causing mutation [20], further emphasising the importance of Rab binding in a physiological context. To further test the functionality of disease-causing mutations in endocytic uptake within the pronephros, we expressed a second Lowe syndrome mutant, L746P, or a Dent-2 disease mutant, P858L (equivalent to human L687P and P799L, respectively [49,50]) and monitored dextran uptake in the pronephros. In both cases the ability to rescue dextran endocytosis was poor (Fig. 6D and E), even though both constructs were expressed at comparable levels to wild-type OCRL1 used in parallel rescue experiments (Fig. 6C). This finding strongly supports the hypothesis that renal tubular endocytosis is defective in both Lowe syndrome and Dent-2 disease.

### Suppression of PIP5K activity rescues renal endocytosis in OCRL1-/- mutant embryos

**Fig 7:**
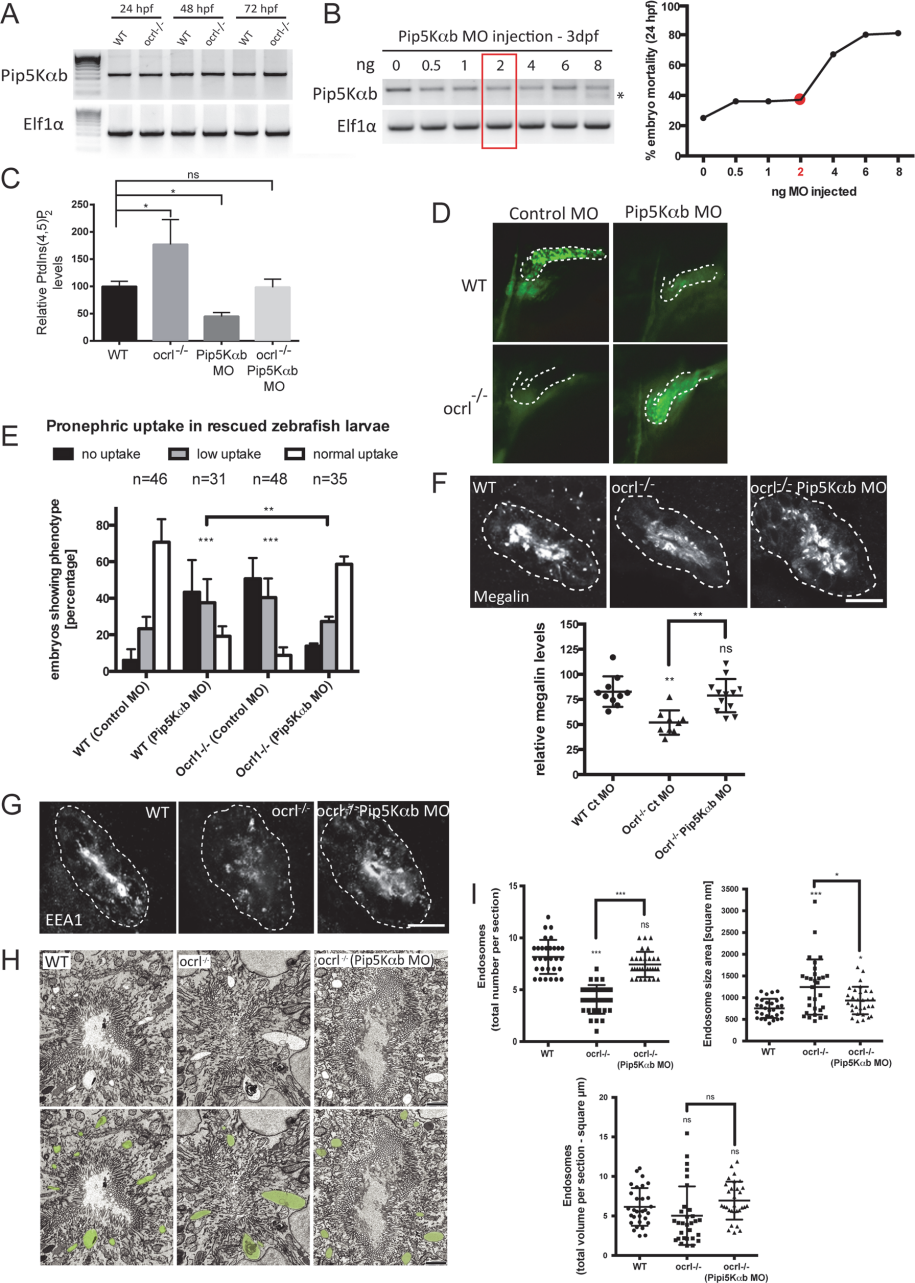
Rescue of the pronephric uptake defect in OCRL1 deficient embryos by suppression of PIP5K. A. RT-PCR detection of PIP5Kαb and eIF1α in wild-type and *ocrl^-/-^* embryos at the indicated developmental timepoints. B, left. RT-PCR of PIP5Kαb and eIF1α in 3 dpf zebrafish embryos injected with the indicated amount of PIP5Kαb splice morpholino. The asterisk indicates morpholino-induced abnormally spliced PIP5Kαb transcript. Right, mortality of PIP5Kαb morpholino-injected embryos at 24 hpf. C. PtdIns(4,5)P_2_ levels in untreated wild-type or *ocrl^-/-^* embryos or embryos injected with 2 ng PIP5Kαb morpholino. Data are presented as the mean ± SE (n = 6–13). Statistical analysis was performed using the one-way ANOVA with a post-hoc Dunnett’s multiple comparisons test. *p < 0.05. D. Images of pronephric uptake of Alexa 488-10 kDa dextran (green) in wild type (WT) or *ocrl^-/-^* embryos or WT or *ocrl^-/-^* embryos injected with 2 ng PIP5Kαb morpholino. The pronephric tubules are indicated with a green dashed line. E. Quantification of pronephric uptake of Alexa 488-10 kDa dextran in each of the indicated embryo types. F. Transverse confocal images showing megalin labelling in the proximal pronephric region of 72 hpf wild-type (WT), *ocrl^-/-^* or *ocrl^-/-^* embryos injected with 2 ng PIP5Kαb morpholino (top) and quantitation of megalin fluorescence (bottom). G. Transverse confocal images showing EEA1 labelling in the proximal pronephric region of 72 hpf wild-type (WT), *ocrl^-/-^* or *ocrl^-/-^* embryos injected with 2 ng PIP5Kαb morpholino. H. Block face scanning electron microscopy images of transverse sections through the proximal pronephric tubule of wild-type (WT), *ocrl^-/-^* or *ocrl^-/-^* embryos injected with 2 ng PIP5Kαb morpholino. The bottom row is a colour-coded version of the top row, with vacuaolar endosomes false coloured in green. I. Quantification of vacuolar endosome number, size and total area. Data in E, F and I are presented as the mean ± SEM. Statistical analysis was performed using the Pearson’s chi-squared test. ***p < 0.0001, **p < 0.001, *p < 0.01. Scale bars represent 10 μm (F, G) and 2 μm (H).

The inability of catalytically inactive OCRL1 to rescue dextran uptake in the pronephros of *ocrl^-/-^* embryos suggests that accumulation of PtdIns(4,5)P_2_ in these embryos, as observed previously [31], results in impaired endocytosis. If so, then it may be possible to restore endocytosis in the *ocrl^-/-^* embryos by rebalancing PtdIns(4,5)P_2_ levels to physiological levels. We therefore sought to inhibit production of PtdIns(4,5)P_2_ in the *ocrl^-/-^* embryos by suppressing expression of PtdIns4P 5-kinase (PIP5K) and test whether endocytosis could be restored. This approach has been used successfully in OCRL1-depleted cultured cells [8]. We identified two zebrafish orthologues of PIP5Kα, the mammalian PIP5K isoform that was most efficient in rescuing mammalian cultured cells [8]. The two zebrafish orthologues have likely arisen through teleost specific gene duplication. We decided to study the PIP5Kαb isoform, which has highest homology to mammalian PIP5Kα, and confirmed its expression through early zebrafish development by RT-PCR (Fig. 7A). The level of PIP5Kαb expression was comparable in wild-type and *ocrl^-/-^* embryos (Fig. 7A). We titred a splice-blocking morpholino to suppress PIP5Kαb expression to varying degrees and monitored embryo viability and renal endocytosis. An intermediate dose of morpholino was found to partially suppress PIP5Kαb expression without affecting embryo viability (Fig. 7B). Measurement of PtdIns(4,5)P_2_ indicated that this dose of morpholino was sufficient to reduce PtdIns(4,5)P_2_ levels when injected into wild-type embryos (Fig. 7C). More importantly, it could rebalance PtdIns(4,5)P_2_ to wild-type levels when injected into the *ocrl^-/-^* mutant, which in the absence of morpholino had elevated levels of PtdIns(4,5)P_2_, as expected (Fig. 7C). The PIP5Kαb morpholino was found to inhibit endocytosis when injected alone into wild-type embryos (Fig. 7D and E), indicating a requirement for PtdIns(4,5)P_2_ in renal tubular endocytosis. Strikingly however, the same degree of PIP5Kαb suppression in the OCRL1-deficient mutant embryos was able to restore endocytosis (Fig. 7D and E). The rescue was efficient, with endocytosis restored to wild-type levels. Moreover, megalin levels and endosome number and morphology were also restored upon PIP5Kαb suppression (Fig. 7F-I). These results indicate that the levels of PtdIns(4,5)P_2_ are critically important for endocytic trafficking in the renal tubule. Importantly, in the case of the *ocrl^-/-^* mutant, endocytosis can be fully rescued by restoring PtdIns(4,5)P_2_ back to physiological levels.

## Discussion

In this manuscript we report that a deficiency of OCRL1 results in impaired endocytosis within the zebrafish pronephric tubule. There is reduced apical uptake of both fluid phase markers and low molecular weight protein from the glomerular filtrate, consistent with a general endocytosis defect. The reduced abundance of early endocytic compartments in OCRL1 deficient embryos supports this conclusion. A role for OCRL1 in endocytic trafficking was proposed a number of years ago [28], but in vitro studies have yielded conflicting results regarding OCRL1 involvement in this process [6,8,9,22,23]. Moreover, whether OCRL1 can regulate trafficking in vivo, in a more physiological context, has not been shown. Our results provide the first evidence that OCRL1 does indeed regulate endocytic trafficking in vivo, and support the hypothesis that defects in this process cause the renal tubulopathy of Lowe syndrome and Dent-2 disease.

Our results are consistent a role for OCRL1 in endocytic recycling within the proximal tubule. Vicinanza et al demonstrated that loss of OCRL1 in cultured cells inhibits recycling of several receptors, including megalin, to the plasma membrane [8], and the OCRL1 interacting proteins IPIP27A and B were also found to function in recycling [21]. The reduced abundance of megalin protein but not transcript in OCRL1 deficient embryos can be explained by reduced recycling. The same phenomenon is seen in a mouse model for Dent-1 disease, where loss of the endosomal chloride transporter CLC5 causes a megalin recycling defect [38,39]. The failure to retrieve megalin from endosomes could result in its delivery to lysosomes and subsequent degradation. However, CLC5-deficient mice are also defective in delivery of megalin to the lysosome [39], indicating that non-lyosomal degradation can occur when megalin recycling is impaired. Hence, further studies will be required to determine how megalin is degraded upon OCRL1 or CLC5 deficiency. The reduced abundance of megalin at the apical membrane that we report here is consistent with the observation that Lowe syndrome and Dent disease patients shed less megalin ectodomain into their urine [28].

Although our findings are most consistent with a recycling defect, we cannot exclude the possibility that OCRL1 may also affect the internalisation stage of endocytosis. It is present in clathrin coated pits at the plasma membrane [7,9,19], and a recent study revealed OCRL1 involvement in late stages of clathrin vesicle biogenesis [23]. However, others have failed to observe a defect in receptor uptake at the cell surface [8,22,51]. Rather, reduced endocytosis is thought to arise from the presence of fewer receptors at the plasma membrane as a consequence of defective recycling [8]. This is most consistent with our in vivo observations. We also cannot formally exclude a role for OCRL1 in the biosynthetic delivery of megalin, as recently suggested for the intestinal calcium channel TRPV6 [52], but its failure to accumulate within the secretory pathway of OCRL1 deficient embryos is inconsistent with such a role.

The loss of early endocytic compartments in the OCRL1 deficient embryos is reminiscent of the situation in megalin knockout mice and zebrafish [40,41], although it is less pronounced. A plausible explanation is that because megalin, the major endocytic receptor in the proximal tubule, is less abundant in these situations, there is less internalisation and delivery of membrane to early endosomes. The reduction in endocytic vesicles in the OCRL1 deficient zebrafish would support this model. It was recently reported that the abundance of endocytic compartments in vivo is critically dependent on the master regulator Rab5 [53]. Our findings and the earlier megalin knockout studies indicate that the abundance of endocytic receptors is also an important factor. We failed to observe changes in the levels of transcripts for various endocytic components including megalin, EEA1 and Rab5 (see S7 Fig.), indicating that the reduction in endocytic compartments we observe is not a consequence of altered gene expression. Thus, we favour the idea that receptor abundance affects endosomal homeostasis by determining the flux of membrane through the pathway.

A defect in megalin trafficking cannot explain all the aspects of the renal tubulopathy in Lowe syndrome and Dent-2 disease including aminoaciduria, hypercalciuria and phosphaturia. Thus, trafficking of transporters localised at the apical and/or basolateral membrane of cells within the proximal tubule is also likely to be affected by loss of OCRL1. This would be consistent with a more generalised defect in endocytic trafficking, as we observe. Interestingly, CLC5 has been shown to play a role in trafficking not only of megalin, but also of the apical Na^+^H^+^ antiporter NHE3, most likely at the level of endocytic recycling [38,54]. NHE3 is important for maintaining sodium-coupled uptake of numerous ions and small molecules, and its reduced levels at the plasma membrane could account for other aspects of the tubulopathy in Lowe syndrome and Dent disease.

Three recent studies have revealed a role for OCRL1 in ciliogenesis [16,17,25], leading to the possibility that the manifestations of Lowe syndrome and Dent-2 disease arise through ciliary defects. We too observed defective cilia formation. However, unlike the previous studies, we failed to observe any defect in renal clearance, nor the presence of renal cysts, pericardial oedema, or a curved body axis [16,17,25], which are indicative of ciliary dysfunction. This may be due to a less severe loss of the protein in our OCRL1 mutant and morphant larvae, in which OCRL1 is depleted by approximately 70% [31]. Because fluid propulsion in the pronephros is unaffected in our mutant and morphant embryos, it is extremely likely that the endocytic defect is distinct from any defect in cilia function. Moreover, it should be remembered that several of the features of ciliopathies are absent in Lowe syndrome and Dent-2 disease. For example, the Lowe and Dent-2 patients lack renal cysts and do not have retinal degeneration or hepatic disease, which are hallmarks of ciliopathies [55,56]. Although one cannot exclude the possibility that cilia dysfunction contributes to the Lowe and Dent-2 phenotype, it is unlikely to be the sole explanation. Rather, our data favour the hypothesis that defective endocytic trafficking is a major factor, certainly for the renal tubulopathy, but also possibly in the nervous system, where neurons are extremely endocytically active.

The inability of catalytically inactive OCRL1 to rescue endocytosis in the *ocrl^-/-^* embryos, coupled with the rescue of endocytosis by suppression of PIP5K, indicate that it is the accumulation of PtdIns(4,5)P_2_ that is detrimental in the renal tubule in vivo. Our results suggest that compounds that can sequester or inhibit the production of PtdIns(4,5)P_2_ will have value as potential therapeutics for Lowe syndrome and Dent-2 disease. Specifically, PIP5K would seem an attractive drug target, while compounds such as PHDM that can sequester PtdIns(4,5)P_2_ [57], may also be worth exploring. Moreover, monitoring renal endocytosis in zebrafish embryos should allow the functional screening of compounds to identify potential drugs for the treatment of Lowe syndrome and Dent-2 disease.

## Materials and Methods

### Antibodies

The antibodies used in this study were goat anti-EEA1 (Santa Cruz Biotechnology, sc-6415), rabbit anti-megalin (kindly provided by Michele Marino, University of Pisa, Italy), mouse anti-acetylated-tubulin (Sigma, T7451), rabbit anti-endofin (Proteintech Europe, 13118-2-AP), mouse 3G8 and α6F anti-NaK ATPase (Developmental Studies Hybridoma Bank). Sheep anti-GFP antibodies were raised in-house. Fluorophore-conjugated secondary antibodies were purchased from Molecular Probes.

### Molecular biology

All constructs were made using standard molecular biology techniques. Full-length OCRL1 isoform a [31] was cloned into pT2KXIGDin-enpep vector (Dr Michael Pack, University of Pennsylvania, USA) for expression in zebrafish pronephric tubules. Point mutations and small deletions were introduced by PCR using the site-directed mutagenesis Quikchange method (Stratagene). mApple-tagged human Rab11a and GFP-tagged zebrafish Rab11 were cloned into pcGlobin for transient expression in zebrafish embryos. Primer sequences for all manipulations are available upon request. All constructs were verified by DNA sequencing. Plasmid encoding GFP under control of the cardiomyosin light chain 2 promoter (*cmlc2*:GFP) was obtained from Dr Adam Hurlstone (University of Manchester, UK).

### Zebrafish strains and husbandry

Zebrafish were raised and maintained at the University of Manchester Biological Services Unit according to the UK Animals Act 1986. The wild type line was of the AB background. The *ocrl^-/-^* mutant line (ZDB-GENO-120531–1) and the ET33-D10 line were described previously [31,34]. Transgenic zebrafish expressing GFP-Rab5c (Tg(h2afx:EGFP-Rab5c)) and GFP-Rab7 (Tg(h2afx:EGFP-Rab7)) were a kind gift from Dr Brain Link (Medical College of Wisconsin, USA) [58].

### Whole mount in situ hybridization

Dioxigenin-UTP-labeled megalin probe was transcribed from linearized plasmid (from Professor Thomas Wilnow, MDC Molecular Medicine, Berlin) using T7 RNA polymerase and the Roche DIG RNA labeling kit [33]. Whole mount in situ hybridization was performed according to Ramirez et al [31].

### RNA isolation, RT-PCR and Q-PCR

Total RNA was isolated from zebrafish embryos using Trizol (Invitrogen) and reverse-transcribed with Superscript First Strand (Invitrogen) to produce cDNA. For direct visualization of amplification products, cDNA was amplified using standard PCR conditions and appropriate primer pairs. Q-PCR was performed using SYBR Green (Sigma-Aldrich) according to the manufacturer’s protocol. 0.5 μl cDNA template from a 20 μl aliquot generated from 5 μg of RNA was used per reaction. Each experiment was run in duplicate and was repeated on three individually obtained RNA extracts. Reactions were performed using ABIPrism 7000 sequence detector system (Applied Biosystems Ltd).

### RNA, DNA and morpholino injections in zebrafish

Capped mRNA encoding tol2 transposase was transcribed from the pCS2-FA vector (Dr Michael Pack, University of Pennsylvania, USA) using the mMessage mMachine kit (Ambion) and approximately 1 nl of a mix of 10 ng/μl pT2KXIGDin-enpep vector containing OCRL1a, 10 ng/μl *cmcl2*:GFP vector and 20 ng/μl tol2 transposase mRNA was injected into one-cell stage mutant embryos. mApple-Rab11a capped mRNA was transcribed using the mMessage mMachine kit (Ambion) and 1 nl of 200 ng/μl was injected into one-cell stage embryos. Morpholinos were obtained from GeneTools. Control and OCRL1 morpholinos were injected as described previously [31]. Morpholino targeting zebrafish PIP5Kαb (ENSDARG00000024642—splice blocking MO: TGCGTGTTATATCTAACAGATAGTC) was injected at 2 ng into one cell stage embryos. The IFT88/Polaris splice blocking MO was described previously [34].

### Injection and analysis of endocytic tracers

Lysine-fixable 10 kDa, 70 kDa or 500 kDa dextran labelled with Alexa 488, TexasRed, or FITC respectively (Molecular Probes) were prepared in PBS at 2 μg/μl final concentration. Recombinant Cy3-labelled His-tagged RAP (39 kDa) was prepared in PBS at 5 μg/μl final concentration. The injected volume was adjusted individually for every dye used based on the total fluorescence in the larvae circulatory system. Zebrafish embryos at 72 hpf were anesthetized with 0.2 mg/ml MS222, (Sigma) in chorion water, and tracer injected into the common cardinal vein using a glass micropipette PLI-90 Pico-Injector (Harvard Apparatus). Pronephric uptake was assessed at 45 min to 4 hours after injection on whole mounts using a fluorescent dissecting stereomicroscope (Leica MZ10F). Statistical analysis was performed using the Pearson’s chi-squared test with Prism software (Prism Software Corporation). Excretion of 10 kDa Alexa 488-dextran from the cloacae was visually monitored in live embryos 30–60 s after injection, and numbers of embryos excreting dextran at similar amounts to control were scored.

### Fluorescence microscopy

Zebrafish embryos were fixed using 4% PFA or Dent’s Fixative and whole-mount labelling performed according to [32]. For cryosectioning embryos were mounted in cryosectioning moulds, frozen on dry ice and sectioned using a Leica CM3050 S cryotome. Images were captured using a Leica SP5 confocal microscope and analysed using ImageJ software. Statistical analysis was performed using the t-test with Prism software (Prism Software Corporation). Megalin fluorescence intensity measurements were performed with ImageJ. The region of interest was selected outlining the periphery of the kidney tubule, and background fluorescence was set at the same intensity as the internal lumen, and subtracted from the total fluorescence intensity. Bright field images of whole embryos were taken on a Zeiss SteREO Lumar V.12 stereomicroscope.

### Electron microscopy

The samples for electron microscopy were processed by the EM Facility at the Faculty of Life Sciences, University of Manchester, UK. The samples were fixed in 2.5% glutaraldehyde + 4% formaldehyde and processed using a high density staining method suitable for serial block face imaging [59]. Samples were embedded in Epon812 (hard formulation) and trimmed on a standard microtome in order to locate the pronephros. The samples were then examined using a Gatan 3view microtome within an FEI Quanta 250 FEG scanning electron microscope. In order to assay a large volume of pronephros at high resolution a script was written using AutoIt v3 to control Gatan Microscopy Suite. The script allowed a large image (7000x7000 pixels with a 10 μs dwell time) of each pronephros to be collected every ten 100 nm cuts. This allowed images with a pixel size of resolution of 10 nm, but sufficiently large (70 μmx70 μm) to track the whole pronephros to be taken every 1 μm. Following acquisition the raw data was converted into 8 bit tifs using IMOD [60]. Images were analysed using ImageJ. Endocytic compartments were defined by morphology. Apical endocytic vesicles are apically localized oval or spherical membrane-enclosed compartments with electron sparse content, of less than 250 nm diameter. Vacuolar endosomes are oval or spherical membrane-enclosed compartments of a diameter greater than 500 nm, with an electron sparse lumen that contains varying degrees of granular material. Electron dense recycling tubules and intralumenal vesicles are frequently but not always observed at these compartments.

### Western blotting

Thirty zebrafish embryos (3 dpf) were homogenized in 60 μl SDS sample buffer by passing 10x through an 18G syringe and heating at 95°C for 5 min. Extracts were clarified by centrifugation prior to SDS-PAGE on a 4% gel followed by Western blotting.

### PtdIns(4,5)P_2_ measurements

Measurement of PtdIns(4,5)P_2_ was performed according to [61] with the only modification being that detection of filter-bound PtdIns(4,5)P_2_ was carried out using recombinant bacterially expressed His-GFP-PLCδ (plasmid kindly provided by Dr Tim Levine, UCL, London) followed by sheep anti-GFP and HRP anti-sheep antibodies.

### Ethics statement

All work was performed under the UK Home Office animal project licence number 70/8132. Local animal care was provided by the University of Manchester BSF Unit. Zebrafish embryos at 72 hpf were anesthetized with 0.2 mg/ml MS222 for injection of endocytic tracers. Following the experiment, euthanasia was performed by incubation in 0.2 mg/ml MS222 for >2 h.

## Supporting Information

S1 Fig Dextran uptake assay and colocalization with endosomes. A. Schematic representation of pronephric tubules (green) in 72hpf zebrafish embryos (left lateral view, top right dorsal view) (top) and pronephric uptake assay in 72hpf zebrafish embryos (bottom) showing embryos immediately after injecting Alexa 488-10 kDa dextran (10kDa-FD, green)(left) or after 2.5h incubation to allow accumulation in the pronephros (arrow) (right). B. Confocal transverse section of zebrafish proximal pronephric tubule of 72 hpf zebrafish embryos injected with Alexa 488-10 kDa dextran (green) and incubated for 2.5 h prior to fixation and labelling with antibodies to the early endosome marker EEA1 (red). Scale bar, 5 μm.
(EPS)

S2 Fig Impairment of pronephric uptake in OCRL1 deficient zebrafish embryos. Fluorescence dissecting microscopy of wild-type (WT), *ocrl^-/-^* mutant, control morphant or OCRL1 morphant 72 hpf zebrafish embryos that were injected with Alexa 488-conjugated 10 kDa dextran (green) and imaged after 2.5h. Dorsal view.
(EPS)

S3 Fig Pronephric tubule development in *ocrl^-/-^* embryos. A. Dorsal view of 33D10-GFP zebrafish embryos (72hpf) expressing GFP in the pronephric tubules injected with control or OCRL1 morpholino (MO) (left). Lateral view of wild type and *ocrl^-/-^* zebrafish embryos (72hpf) expressing *enpep*:GFP in the pronephric tubules (right). B. Confocal images of pronephric tubules of wild-type (WT), *ocrl^-/-^* mutant, control morphant or OCRL1 morphant 72 hpf zebrafish labelled with the 3G8 anti-brush border antibody (dorsal view).
(EPS)

S4 Fig Pronephric filtration of 500 kDa-FD. A. Fluorescence dissecting microscope image of zebrafish embryos (72hpf) injected with 500 kDa dextran conjugated with FITC (500 kDa-FD) immediately after injection (top) and after 24h: wild type (middle) and *ocrl^-/-^* embryos (bottom) (96hpf). Retained 500 kDa-FD (green) is present in the vasculature of both embryo types.
(EPS)

S5 Fig OCRL1 deficiency does not affect cell polarity. A. Confocal transverse sections of the zebrafish proximal pronephric tubule of 72 hpf wild-type (WT), *ocrl^-/-^* mutant, control morphant or OCRL1 morphant embryos labelled with anti-brush border (3G8, green) and anti-megalin (red) antibodies. B. Confocal transverse sections of the zebrafish proximal pronephric tubule of 72 hpf wild-type (WT), *ocrl^-/-^* mutant, control morphant or OCRL1 morphant larvae labelled with anti-NaK ATPase (green) and anti-megalin (red) antibodies. Scale bars represent 5 μm.
(EPS)

S6 Fig Brush border and intercellular junctions of *ocrl^-/-^* zebrafish pronephric cells. A. Block face scanning electron microscopy images of microvilli at the apical brush border of pronephric tubule cells of wild type and *ocrl^-/-^* embryos (72hpf). B. Transmission electron microscopy images of intercellular junctions between pronephric cells of wild type and *ocrl^-/-^* embryos (72hpf). AJ = adherent junctions, TJ = tight junctions, DS = desmosomes. Scale bars represent 0.5 μm (A) and 100 nm (B).
(TIF)

S7 Fig Pronephric cilia in *ocrl^-/-^* zebrafish. A. Fluorescence dissecting microscope image of wild-type (WT) and OCRL1-/- mutant zebrafish embryos (26hpf) labeled with anti-acetylated-tubulin antibody (top, pronephric cilia are indicated with arrows, lateral view). Confocal images of pronephric cilia in wild-type (WT), *ocrl^-/-^* mutant, control morphant or OCRL1 morphant zebrafish embryos (26hpf) (bottom). B. Fluorescence dissecting microscope image of dextran excretion from the cloacae of zebrafish embryos (72hpf). Bottom panels show cloacae immediately after injection (left) and excreting dextran (arrows) 30–60s after injection (wild-type middle, *ocrl^-/-^* right). C. Confocal transverse sections of the zebrafish proximal pronephric tubule of 72 hpf wild type and *double bubble (dbb*) cilia mutant showing 10 kDa-FD uptake (red) in pronephric cells 2h after injection. Scale bars represent 10 μm.
(EPS)
