# Supplementary figures and images for: The Lowe Syndrome Protein OCRL1 Is Required for Endocytosis in the Zebrafish Pronephric Tubule
Source: PLoS Genet. 2015 Apr 2;11(4):e1005058. doi: 10.1371/journal.pgen.1005058 (PMC4383555; doi:10.1371/journal.pgen.1005058)

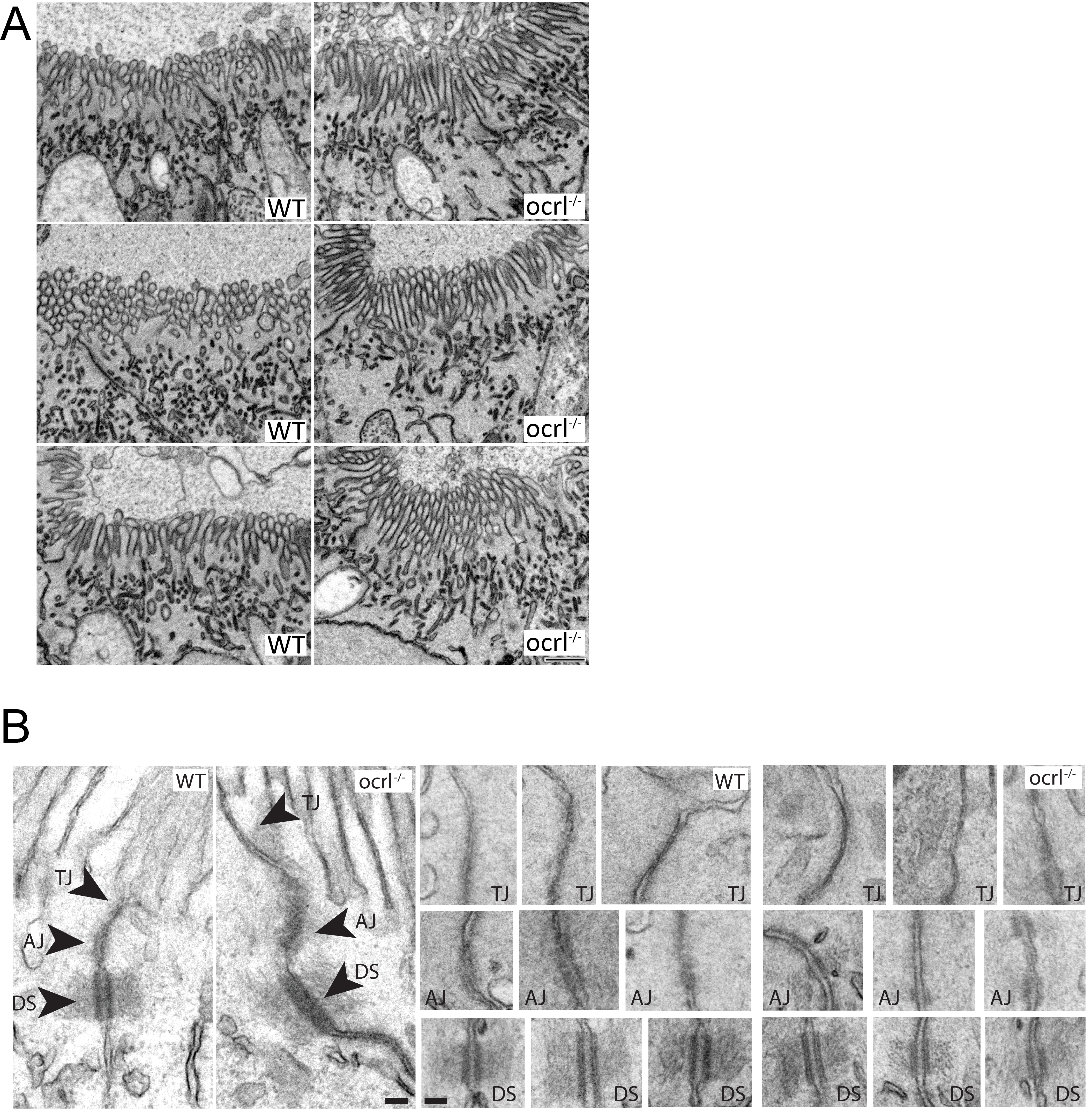

Supplement: S6 Fig — A. Block face scanning electron microscopy images of microvilli at the apical brush border of pronephric tubule cells of wild type and ocrl-/- embryos (72hpf). B. Transmission electron microscopy images of intercellular junctions between pronephric cells of wild type and ocrl-/- embryos (72hpf). AJ = adherent junctions, TJ = tight junctions, DS = desmosomes. Scale bars represent 0.5 μm (A) and 100 nm (B). (TIF) [file pgen.1005058.s006.tif]
